# Prognostic Factors for Mortality in Hepatocellular Carcinoma at Diagnosis: Development of a Predictive Model Using Artificial Intelligence

**DOI:** 10.3390/diagnostics14040406

**Published:** 2024-02-13

**Authors:** Pablo Martínez-Blanco, Miguel Suárez, Sergio Gil-Rojas, Ana María Torres, Natalia Martínez-García, Pilar Blasco, Miguel Torralba, Jorge Mateo

**Affiliations:** 1 Gastroenterology Department, Virgen de la Luz Hospital, 16002 Cuenca, Spain; 2 Medical Analysis Expert Group, Institute of Technology, Universidad de Castilla-La Mancha, 16071 Cuenca, Spain; 3 Medical Analysis Expert Group, Instituto de Investigación Sanitaria de Castilla-La Mancha (IDISCAM), 45071 Toledo, Spain; 4 Internal Medicine Unit, Guadalajara University Hospital, 19002 Guadalajara, Spainmiguel.torralba@uah.es (M.T.); 5 Department of Pharmacy, General University Hospital, 46014 Valencia, Spain; 6 Faculty of Medicine, Universidad de Alcalá de Henares, 28801 Alcalá de Henares, Spain; 7 Translational Research Group in Cellular Immunology (GITIC), Instituto de Investigación Sanitaria de Castilla-La Mancha (IDISCAM), 45071 Toledo, Spain

**Keywords:** hepatocellular carcinoma, machine learning, prognosis, mortality, Milan criteria, BCLC, albumin

## Abstract

Background: Hepatocellular carcinoma (HCC) accounts for 75% of primary liver tumors. Controlling risk factors associated with its development and implementing screenings in risk populations does not seem sufficient to improve the prognosis of these patients at diagnosis. The development of a predictive prognostic model for mortality at the diagnosis of HCC is proposed. Methods: In this retrospective multicenter study, the analysis of data from 191 HCC patients was conducted using machine learning (ML) techniques to analyze the prognostic factors of mortality that are significant at the time of diagnosis. Clinical and analytical data of interest in patients with HCC were gathered. Results: Meeting Milan criteria, Barcelona Clinic Liver Cancer (BCLC) classification and albumin levels were the variables with the greatest impact on the prognosis of HCC patients. The ML algorithm that achieved the best results was random forest (RF). Conclusions: The development of a predictive prognostic model at the diagnosis is a valuable tool for patients with HCC and for application in clinical practice. RF is useful and reliable in the analysis of prognostic factors in the diagnosis of HCC. The search for new prognostic factors is still necessary in patients with HCC.

## 1. Introduction

Primary liver tumors are the seventh most common cause of cancer worldwide and the second leading cause of cancer-related deaths. Hepatocellular carcinoma (HCC) is the most common primary liver neoplasm, accounting for 75% of cases of primary liver malignancies [1]. HCC is an adenocarcinoma-type neoplasm that originates in hepatocytes due to sustained cellular damage and stress. This damage induces chronic inflammatory changes, necrosis, and fibrosis in hepatocytes, promoting the development of advanced liver disease (ACLD) [2]. The development of advanced chronic liver disease (ACLD) of any etiology is a key factor for HCC development. Up to 80% of HCC cases occur in livers with ACLD. Viral infections with hepatitis C virus (HCV) and hepatitis B virus (HBV), metabolic dysfunction-associated steatotic liver disease (MASLD), and alcohol consumption are the major determinants in the development of ACLD and HCC [3,4]. The prognosis for patients diagnosed with HCC remains poor, with a global mortality rate of 8.3 per 100,000 individuals in 2020 [5]. These data underscore the need for further research in this field.

HCC is diagnosed through imaging tests such as computed tomography (CT), hepatic magnetic resonance imaging (MRI), or contrast-enhanced ultrasound (CEUS) in patients with ACLD or chronic infection with HBV. Currently, confirmatory liver biopsy is not always necessary [6,7], reserved for cases of uncertain diagnosis and for conducting a more detailed histological analysis that allows for molecular and immunological studies to guide treatment, especially in the context of immunotherapy [8,9,10]. Factors such as tumor burden, liver dysfunction, and the patient’s functional status play a crucial role in prognosis and therapeutic approach. Staging systems such as the Barcelona Clinic Liver Cancer (BCLC) evaluate these aspects, along with objective scores like the Model for End-stage Liver Disease (MELD), the albumin–bilirubin (ALBI) score, and biomarkers such as alpha-fetoprotein (AFP) [11,12]. Early detection of HCC is associated with increased survival [13], and screening programs based on ultrasound monitoring and AFP measurements every 6 months are implemented in patients with ACLD or chronic HBV infection [3,14,15,16]. However, the precise identification of risk populations remains a challenge, and scores like PAGE-B and GALAD assist in risk stratification [17,18]. Despite advances, accurate risk prediction, early detection, prognosis at diagnosis, and personalized treatments are areas that require further research and development [19]. Improving studies on prognostic factors for mortality in HCC is essential to determine their impact on clinical decision making.

Considering the limited published scientific literature, the following study assessing clinical and analytical data for HCC diagnosis was proposed. The main goal is to develop a predictive model for prognostic factors at the diagnosis of this type of tumor. For this purpose, ML algorithms are used. ML employs statistical and mathematical algorithms to extract patterns from variable data, assisting in making complex decisions in medical applications [20,21,22]. These models are designed to make accurate predictions using data from a multitude of variables. In this study, the random forest (RF) algorithm was used. The selection of this ML method is based on its high accuracy, versatility, applicability in large datasets, ability to estimate variable importance, and the lesser need for tuning compared to other ML methods [23,24]. In this way, a predictive model that enables the evaluation of different variables with the most influence on the mortality and prognosis of our HCC patients has been developed.

## 2. Materials and Methods

### 2.1. Study Design and Population

A multicenter retrospective study was conducted involving patients diagnosed with HCC in two different hospitals in Castilla-La Mancha, Spain. The participating hospitals were the University Hospital of Guadalajara and the Virgen de la Luz Hospital in Cuenca. Data collection spanned from January 2008 to December 2022. The study approval was granted by the research ethics committee of the University Hospital of Guadalajara.

Inclusion criteria were as follows: Diagnosis of HCC through validated imaging techniques or histological examination in cases of uncertain diagnosis in patients aged 18 years and older. Diagnosis through imaging techniques was performed using triphasic CT or MRI, where the characteristic uptake of HCC was observed. Patients whose HCC diagnosis was made at another center where clinical and analytical data for the diagnosis were not collected were excluded. Additionally, patients diagnosed at these centers without available study target variables were also excluded.

### 2.2. Data Collection

Patient general information was collected, including gender; active smoking habits at the time of diagnosis; age at HCC diagnosis; censoring date, or date of death if it occurred; and the etiology attributed to HCC (alcohol, HCV, HBV, MASLD, hemochromatosis, autoimmune hepatitis, etc.).

Important clinical characteristics in patients with HCC were noted: the presence of cirrhosis, ACLD, encephalopathy, or clinically significant portal hypertension (CSPH). CSPH was defined as the presence of a hepatic venous portal pressure gradient (HVPG) > 10 mmHg, ascites, or the presence of varices during gastroscopy [25]. Other critical data in tumor assessment, such as the presence of lymphadenopathy, metastasis, portal thrombosis, number and size of hepatic lesions, were also recorded.

Important hepatic analytical data, including albumin (g/dL), international normalized ratio (INR), alanine aminotransferase (ALT) (U/L), aspartate aminotransferase (AST) (U/L), AFP (ng/mL), total bilirubin (mg/dL); other relevant general biochemical data like creatinine (mg/dL), sodium (Na) (mmol/L), C-reactive protein (CRP) (mg/L); and hematologic information involving lymphocyte count (cell/mmc), neutrophil count (cell/mmc), and platelet count (cell/mmc) were also collected.

Using the collected information, staging indices, liver function, and patient baseline status were calculated, including BCLC, Milan Criteria, Child–Pugh, MELD, ECOG (Eastern Cooperative Oncology Group), and TNM. These variables have been validated as useful tools in the management and assessment of patients with liver disease and cancer [11,26,27,28].

### 2.3. Model Development

To conduct the data analysis, the use of ML techniques was proposed. Specifically, the RF algorithm was chosen to develop the predictive model.

The RF algorithm was compared with other ML methods to verify its accuracy. The algorithms used for comparison were support vector machine (SVM), Bayesian linear discriminant analysis (BLDA), decision tree (DT), Gaussian naïve Bayes (GNB), and K-nearest neighbors (KNN).

SVM is a supervised learning algorithm designed for classification. They seek an optimal hyperplane in a higher-dimensional space, maximizing the margin between classes. SVM handles non-linear data using the kernel trick, transforming it into a more manageable space [24,29].

BLDA extends linear discriminant analysis (LDA) with additional probabilistic assumptions. It assumes multivariate normal distribution within each class and employs Bayesian approaches. BLDA is particularly useful when classes exhibit different distributions or varying variances [24,30].

DT is a predictive model structured as trees featuring decision rules and outcomes. Nodes include the root, internal nodes, and leaf nodes. Depth impacts model generalization, and pruning is applied to prevent overfitting. Construction involves recursively selecting features to split data, maximizing homogeneity [24,31].

GNB is a variant assuming Gaussian distribution for input features. Widely used in classification, it requires a training dataset with class-labeled examples. Parameters for Gaussian distribution are calculated for each class, and Bayes’ rule is used for classification, providing a probabilistic estimation [24,32].

KNN is a supervised learning algorithm for classification based on the majority of labels from k-nearest neighbors. It relies on a training dataset with labeled examples, utilizing a chosen distance metric and a specified k value. Classification involves voting among the k neighbors to determine the label for a new point [33].

Among the various ML techniques, the decision to use this algorithm was made due to its interesting features in this study. On one hand, the RF algorithm is an ensemble learning model. It combines multiple models to obtain a more robust and accurate model compared to a single model. The foundation of RF lies in decision trees. Several decision trees are built during the training process. “Randomness” is key in Random Forest. Variations are introduced during the construction of each tree, either through the random selection of features or through the random selection of samples from the dataset. A process called “bagging” (Bootstrap Aggregating) is used to build the trees in the RF model. This involves training each tree with a random sample from the dataset, allowing the trees to be different from each other. RF is known for being robust against overfitting and for handling large datasets with many features [23,24,34]. This technique is widely used in practice due to its good performance and versatility [35].

Primarily, RF employs an ensemble of decision trees. Each tree is trained independently, and their results are then combined. For RF, the trees are constructed in parallel and independently of each other [24,36].

**Figure 1:**
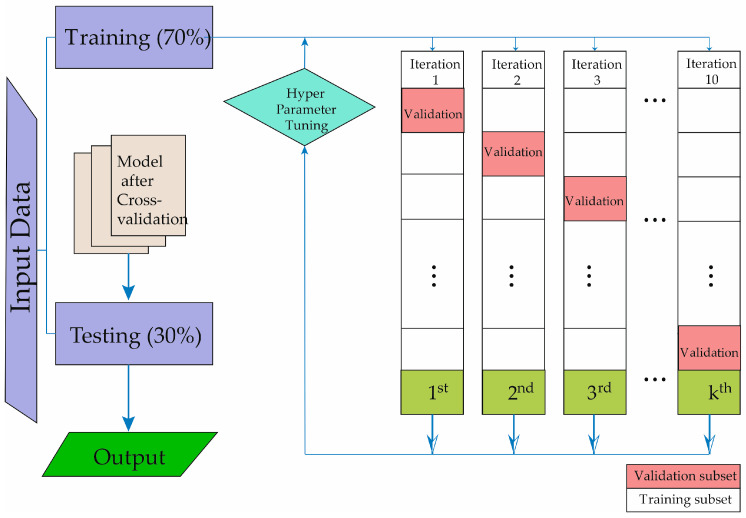
Description of the machine learning methodology development process.

In Figure 1, the technique of cross-validation is described for obtaining our predictive model, allowing the development of machine learning. The study data are divided into two groups. The evaluation cohort of patients underwent 10-fold cross-validation to assess the algorithm’s performance. In each validation fold, 70% of the patients were utilized for training, while the remaining 30% were allocated for testing and validation. The testing process was iterated a hundred times, each time with non-overlapping patient subsets. To prevent the algorithm from being tested on data from the same patients used for training, patient data were not shared between the training and testing subsets. Figure 1 illustrates the step-by-step process undertaken for the entire study. As depicted, the initial phase involved the selection of subjects to be studied. Following the creation of the database, the machine learning methods were then trained and validated.

ML techniques typically incorporate one or more hyperparameters that facilitate the adjustment of the algorithm throughout the training process. Varied values for these hyperparameters, such as the number of splits, learners, neighbors, distance metric, distant weight, kernel, box constraint level, and the multiclass method, among others, contribute to distinct prediction performances. The objective is to achieve optimal performance by optimizing these hyperparameters for each machine learning technique utilized in this study. Bayesian optimization was employed for hyperparameter tuning, aiming to identify configurations that maximize algorithm performance based on previous attempts. This approach assumes a relationship between different hyperparameters and algorithm performance. Performance measures, specifically the area under the curve (AUC) and balanced accuracy were employed for maximization.

The most prominent hyperparameters of the implemented systems are as follows. For the SVM method, a Gaussian kernel function is chosen with the following parameters: C = 1, sigma = 0.5, numerical tolerance = 0.001, and iteration limit = 100. For the DT system, the base parameter estimator is adjusted: tree, maximum number of splits = 20, learning rate = 0.1, and number of learners = 40. GNB algorithm: usekernel: False, fL = 0, and Adjust = 0. As for the BLDA algorithm, the Bayesian kernel was selected. Finally, for the KNN method, the distance metric was Euclidean, and it used 20 neighbors.

### 2.4. Performance Evaluation

In this work, the different methods were compared with the following metrics: specificity, precision (also known as positive predictive value), recall (also known as sensitivity), balanced accuracy, degenerate Youden index (*DYI*), *F*_1_ *score* Matthew’s correlation coefficient (*MCC*), Cohen’s Kappa index (CKI), receiver operating characteristic (ROC), and area under the curve (AUC) [24].

(1) $$\mathrm{Precision}=\frac{\mathrm{TP}}{\mathrm{TP}+\mathrm{FP}}$$

(2) $$\mathrm{Recall}=\frac{\mathrm{TP}}{\mathrm{TP}+\mathrm{FN}}$$

(3) $$\mathrm{Specificity}=\frac{\mathrm{TN}}{\mathrm{FP}+\mathrm{TN}}$$

(4) $$\mathrm{Accuracy}=\frac{\mathrm{TP}+\mathrm{TN}}{\mathrm{TP}+\mathrm{TN}+\mathrm{FP}+\mathrm{FN}}$$

The *F*_1_ *score* is described as:

(5) $$F_{1}\;\mathrm{score}=2\frac{\mathrm{Precision}\cdot \mathrm{Recall}}{\mathrm{Precision}+\mathrm{Recall}}$$

*MCC* and *DYI* were also used to test the performance of the ML methods, defined as:

(6) $$\mathrm{MCC}=\frac{\mathrm{TP}\cdot \mathrm{TN}-\mathrm{FP}\cdot \mathrm{FN}}{\sqrt{\left(\mathrm{TP}+\mathrm{FP}\right)\left(\mathrm{TP}+\mathrm{FN}\right)\left(\mathrm{TN}+\mathrm{FP}\right)\left(\mathrm{TN}+\mathrm{FN}\right)}}$$

(7) $$\mathrm{DYI}=\sqrt{\left(\mathrm{Recall}*\mathrm{Specificity}\right)}$$

where *TP* shows the number of true positives, *FP* represents the number of false positives, *TN* is the number of true negatives, and *FN* corresponds to the number of false negatives. CKI was used to estimate the overall performance of the system [24].

## 3. Results

In accordance with the mentioned inclusion and exclusion criteria, analyzed data from 191 patients were included.

Baseline data are presented in Table 1 and Table 2, where 86.91% of the patients were males, and 87.96% of the total had ACLD. The mean age at HCC diagnosis was 67.13 years old. Regarding the etiology attributed to HCC, 32.46% were associated with alcohol, 29.32% with HCV, and 13.61% with both. At the time of diagnosis, according to the BCLC classification, 37.17% were in stage A, 30.37% in stage C, 15.18% in stage B, 12.04% in stage D, and 5.24% in stage 0. At diagnosis, 37.17% of the patients met the Milan Criteria. It is noteworthy that only 47.12% of the patients were diagnosed through screening. The median, Q1 (first quartile), Q3 (third quartile), and interquartile range of the age at diagnosis and the most relevant analytical data are documented in Table 2.

Below, the results obtained with the methods in the training phase are presented. As shown in Table 3 and Table 4, the ML models achieve good accuracy in the training phase. The crucial aspect of the training phase is to ensure that the system has the ability to generalize and does not exhibit overfitting. To achieve this, we use the cross-validation technique depicted in Figure 1. This helps prevent the model from memorizing the data and being unable to make accurate predictions on new data. Generalization capacity in machine learning refers to the model’s ability to make accurate predictions on new data, i.e., situations not included in the training set. Successful generalization is essential for a model to be effective in real-world scenarios and to avoid overfitting to specific patterns in the training set [24].

**Figure 2:**
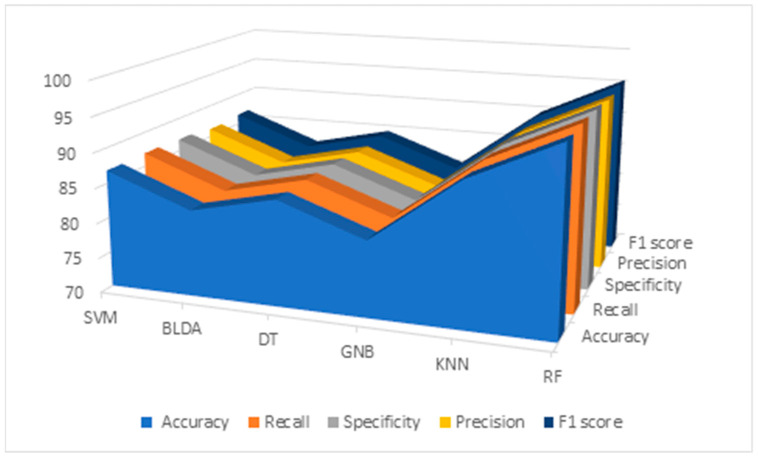
Descriptive figure of performance metrics (accuracy, recall, specificity, precision, and *F*_1_ *score*) for all methods (SVM, BLDA, DT, GNB, KNN, and RF). SVM: support vector machine. BLDA: Bayesian linear discriminant analysis. DT: decision tree. GNB: Gaussian naïve Bayes. KNN: K-nearest neighbors. RF: random forest.

Results of the data analysis conducted by RF and different ML algorithms mentioned before —SVM, BLDA, DT, GNB, and KNN—are presented in Table 5 and Figure 2. As observed, the GNB and BLDA algorithms exhibit the lowest degree of accuracy, just surpassing 80%. KNN demonstrates the highest accuracy, around 90%, but this is still inferior to the one achieved by RF, which reaches 95%. These findings are consistent when analyzing recall, precision, and the *F*_1_ *score* were analyzed.

**Figure 3:**
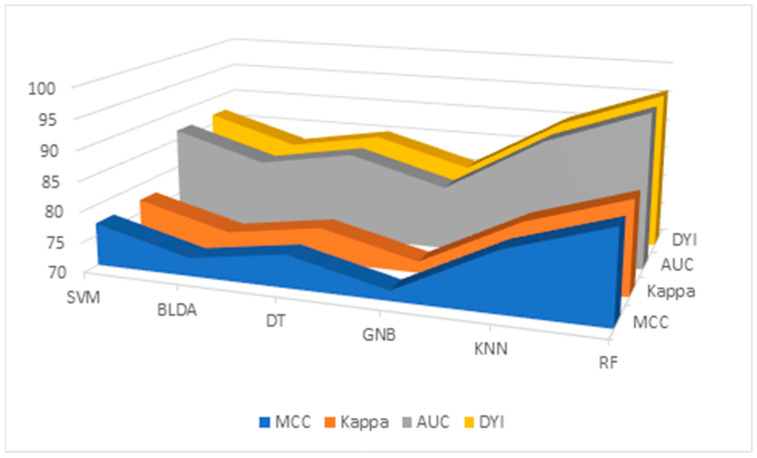
Descriptive figure of performance metrics (MMC, Kappa, AUC, and *DYI*) for all methods (SVM, BLDA, DT, GNB, KNN, and RF). SVM: support vector machine. BLDA: Bayesian linear discriminant analysis. DT: decision tree. GNB: Gaussian naïve Bayes. KNN: K-nearest neighbors. RF: random forest. MMC: Matthews’ correlation coefficient, AUC: area under the curve, *DYI*: degenerated Youden index.

Additionally, the results for the MMC, AUC, *DYI*, and Kappa index are given in Table 6 and Figure 3, for all the proposed methods. GNB shows the lowest results, with an *MCC* of 71.64, *DYI* of 80.73, and Kappa of 71.88. Our proposed RF system exhibits an *MCC* of 85.13, *DYI* of 95.94, and Kappa of 85.41, clearly outperforming the other algorithms, such as KNN, SVM, DT, and BLDA, which have lower values. These results, along with those presented in Table 3, indicate that the RF algorithm, among those proposed in ML, was the most suitable algorithm for data analysis and the development of the proposed predictive model.

**Figure 4:**
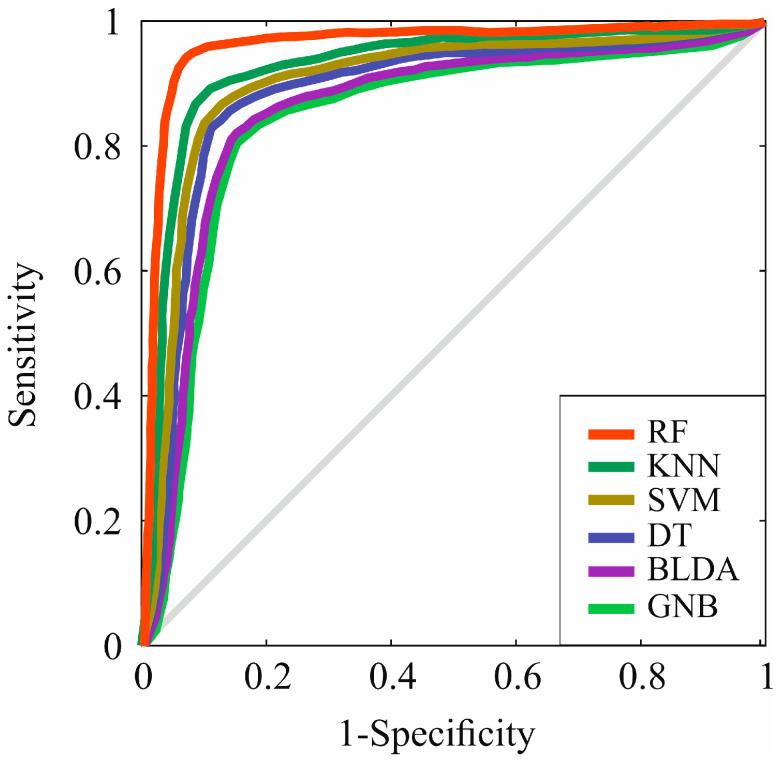
Representation of ROC curves of the analyzed algorithms. RF: random forest. KNN: K-nearest neighbors. SVM: support vector machine. DT: decision tree. BLDA: bayesian linear discriminant analysis. GNB: Gaussian naïve Bayes.

However, in Figure 4, the receiver operating characteristic (ROC) curve of the different ML algorithms can be observed, comparing them among themselves and with the proposed method, RF. As can be observed, in all of them, the AUC is greater than 0.8, but RF presents the highest AUC value compared to the other evaluated methods, standing at 0.96, as shown in Table 6. These results suggest that the RF method has the highest accuracy for predicting prognostic factors at the diagnosis of HCC.

**Figure 5:**
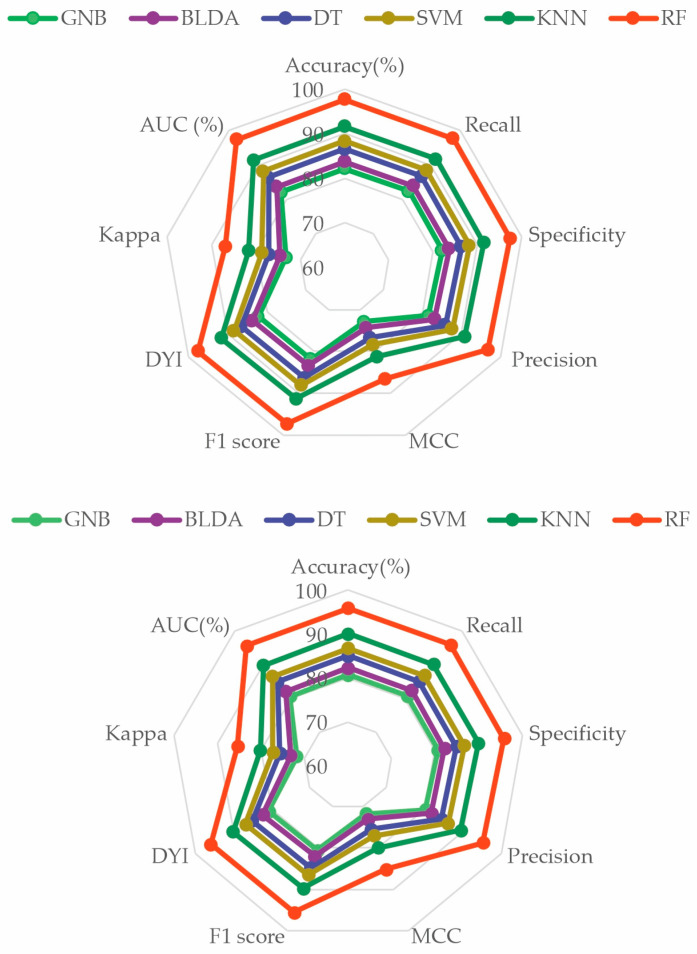
Radar plot comparing all the analyzed methods. The image above is the training phase, and the image below is the test phase. GNB: Gaussian naïve Bayes. BLDA: Bayesian linear discriminant analysis. DT: decision tree. SVM: support vector machine. KNN: K-nearest neighbors. RF: random forest. *MCC*: Matthews correlation coefficient. *DYI*: degenerated Youden index. AUC: area under the curve.

To perform a comprehensive evaluation of all the proposed ML methods, a radar plot was chosen, as depicted in Figure 4. It captures the different metrics and their values for each algorithm. Thus, the larger the area of the circle forming the radar plot, the better the chosen predictive model will be. It is evident that the RF method achieved the widest area. The consistency of the image obtained in both phases—the training phase and the validation phase—demonstrates that RF is accurate, does not overestimate, and therefore, is generalizable. This translates to obtaining appropriate and reliable information with new input data. These results indicate that the RF method is a valid, reproducible, and applicable approach in the development of clinical practice (Figure 5).

**Figure 6:**
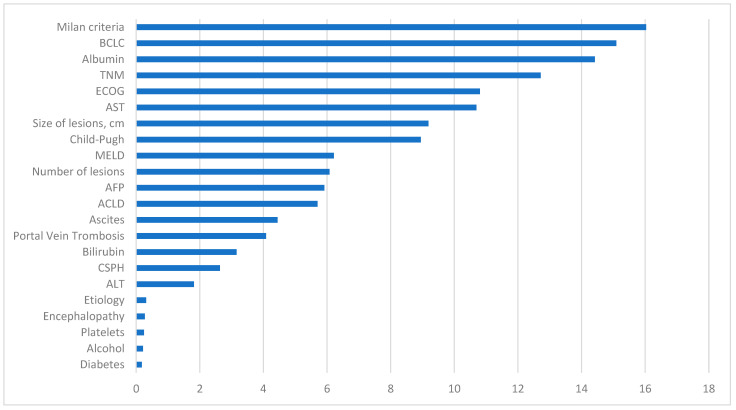
Representation of the importance of each variable in the machine learning predictive model. BCLC: Barcelona Clinic Liver Cancer, TNM: tumor node metastases, ECOG: Eastern Cooperative Oncology Group, AST: aspartate aminotransferase, MELD: model for end-stage liver disease, AFP: alpha-fetoprotein, ACLD: advanced chronic liver disease, CSPH: clinically significant portal hypertension, ALT: alanine aminotransferase.

In Figure 6, the variables with the most significant impact on the development of the predictive model are observed. The fulfillment of Milan Criteria was the most crucial variable, followed by one of the calculated scores, the BCLC classification. Additionally, analytical values such as albumin levels were positioned as the third most important factor in terms of significance. The receipt or non-receipt of a liver transplant, ECOG classification, TNM, hepatic lesions size, Child–Pugh classification, and AST levels also play a substantial role in our predictive model.

Conversely, variables with diminished influence include diabetes status and alcohol consumption. Other analytical data that appear less relevant are platelet count and ALT levels. The etiology attributed to HCC does not exhibit a significant impact in this model, similar to the diagnostic method.

Other initially intriguing markers that did not appear to have a significant influence in our model include CSPH, bilirubin levels, the presence of portal thrombosis, ascites, ACLD, encephalopathy, or AFP levels.

## 4. Discussion

As previously mentioned, liver tumors stand out as neoplasms with a dismal prognosis, currently ranking as the second leading cause of cancer-related deaths [1]. Urgent research in this field is essential to achieve more substantial goals and develop tools that enhance the prognosis of our patients. A targeted approach to address this issue involves intervening in the primary risk factors for the development of HCC and ACLD. Both HCV and HBV emerge as the primary risk factors for ACLD globally. Vaccination campaigns against HBV and effective treatments against HCV have contributed to a reduction in infections caused by these viruses [3]. However, factors such as MASLD and alcohol consumption continue to be determining factors in the development of ACLD and HCC, with an increasing incidence in recent years, particularly in Western countries [3,37]. Indeed, it seems that managing risk factors is advantageous but not entirely sufficient.

Another tool used to attempt to improve the prognosis of patients with HCC is the implementation of screenings in the selected high-risk population [13,38]. The goal is to achieve early diagnosis in patients predisposed to HCC for better management. It has been demonstrated that ultrasound monitoring, along with AFP measurements, appears to be a cost-effective tool in surveilling HCC development [39].

The management of patients with HCC should be coordinated. Multidisciplinary committees led by gastroenterologists, hepatobiliary surgeons, radiologists, oncologists, and radiotherapists work collaboratively. In these committees, the best therapeutic decisions are reached by consensus, aiming to enhance the prognosis of patients with HCC [40,41,42].

Despite applying these tools, the prognosis for these patients remains very limited, necessitating ongoing research and the development of tools to achieve more ambitious goals with these patients.

This study demonstrates that the variables with the most significant impact on the prognosis of patients with HCC are the indices calculated based on clinical and analytical data, such as the Milan criteria. The Milan criteria are used to assess HCC patients eligible for liver transplantation. Patients with a single lesion between 1 and 5 cm or two or three lesions between 1 and 3 cm are considered suitable for transplantation [43]. Consequently, meeting transplant criteria will have a substantial impact on the prognosis of patients with HCC.

This makes sense, as the survival of patients who undergo transplantation is higher than those who are not candidates. Therefore, there has been a proposal to expand the Milan criteria for years. Criteria such as the University of California, San Francisco (UCSF) criteria, up to seven criteria, extended Toronto criteria, and Kyoto criteria evaluate liver transplantation in patients with a higher tumor burden than the limit established by the Milan criteria. It has been demonstrated that the benefit of liver transplantation remains superior in these patients compared to other therapeutic options [44,45]. Another tool used to optimize initially non-transplantable patients is downstaging. It involves administering neoadjuvant local therapy to these patients so that they meet the necessary criteria for liver transplantation [46,47].

The BCLC, as a stratification tool based on tumor burden, the patient’s baseline state, and liver function, also holds substantial significance as a determinant in the prognosis diagnosis of patients in this study. This aligns with the importance demonstrated by other variables that determine tumor burden, such as TNM, or variables that assess the patient’s baseline state, like the ECOG scale [26,28]. This suggests that the combination of both variables, as assessed by BCLC, along with the analytical analysis of other factors, appears to be a suitable option in assessing the prognosis of patients with HCC. Clinical and analytical scores that assess liver function in patients with ACLD, such as the Child–Pugh score, also show significant relevance in the predictive model [27].

Albumin levels were the analytical value of utmost importance in the predictive model. It can be affirmed that albumin levels are determinant as a prognostic factor in patients with HCC. Low levels of serum albumin are associated with poor nutritional status and a worse prognosis at the diagnosis of HCC [48,49]. Furthermore, various studies describe the importance of albumin as a negative regulator in the progression of HCC, particularly in local invasion and metastasis of HCC [50]. It has been demonstrated that albumin levels allow for an adequate assessment of hepatic functional reserve in patients with HCC. They have been combined in different indices such as ALBI or PALBI (platelet–albumin–bilirubin ratio), which discriminate the survival of patients with HCC [51]. In contrast, in this study, the significance of bilirubin levels and platelet count is not as pronounced as that observed with albumin. Further research is needed to evaluate these indices as prognostic factors in HCC.

Nevertheless, ACLD itself and its typical characteristics, such as the presence of ascites, encephalopathy, and CSPH, lack a decisive impact in the predictive model. As mentioned, around 80% of HCC cases occur in the liver with ACLD, and moreover, the population in this study has a similar prevalence, reaching 87.96%. These data suggest that the isolated presence of ascites, encephalopathy, or CSPH does not influence the prognosis of HCC. In contrast, the combination of these factors translates into hepatic dysfunction that does affect the predictive model. It can be concluded that the degree of tumor burden and the patient’s functional status carry more weight than the level of hepatic dysfunction in the prognostic assessment of the study’s patients, highlighting the need for further research.

According to the study findings, not meeting Milan criteria, being in stages B, C, and D of the BCLC, and having low levels of albumin are associated with an unfavorable prognosis in patients with HCC. These data enable us to identify which patients will have a more favorable prognosis and which ones could benefit from different therapeutic options, leading to an increasingly personalized management approach for patients. In this regard, a tool using the proposed method could be generated, and an executable interface could also be installed on medical equipment. This hardware/software tool must comply with European regulations on medical devices and must also have minimum technical characteristics (it is worthwhile to mention that currently, all computers can easily meet these requirements).

An active search was conducted of the current literature on determining prognostic factors for mortality at the diagnosis of HCC using ML techniques. Only one article, authored by Hiraoka et al. [52], was found in which artificial intelligence is utilized to develop a predictive prognostic model related to short- and medium-term survival outcomes. This model is designed to inform decision making regarding the optimal therapeutic approach for both initial and recurrent cases of HCC. Studies that employed ML techniques were identified to classify patients benefiting from immunotherapy in HCC [53] or outlined the application of ML techniques in analyzing radiological and histological images to obtain diagnostic and prognostic information for HCC patients [54]. Within this context, multiple articles describe the analysis of potential prognostic biomarkers for HCC [55,56,57,58,59], which are covered in Piñero et al.’s review [59]. Stefano et al. perform a review of the most relevant biomarkers in the prognosis of HCC [57]. Lima et al. detail, in their review, the potential new minimally invasive biomarkers in the diagnosis and prognosis of HCC [58]. The data analysis in these studies is conducted through conventional statistics, and the review concludes that more tools are needed to determine the prognosis of patients with HCC [59].

The use of ML techniques for the analysis of clinical and analytical data in HCC patients to develop a predictive mortality model and identify prognostic factors at the diagnosis has not been explored. Thus, the aim of this study is to employ various widely used machine learning methods in the scientific and medical community to develop a useful tool for assessing the prognosis of patients with HCC. The selection of the proposed RF method over alternative machine learning algorithms is grounded in the notable advantages that position it as a superior choice in terms of accuracy, robustness, and versatility. Compared to SVM, RF exhibits a unique ability to handle complex and high-dimensional datasets without compromising computational efficiency. The inherent diversity in its ensemble approach minimizes the risk of overfitting, providing more general and predictive models, particularly in scenarios with elevated problem complexity. Against GNB, RF stands out for its effective handling of irrelevant or noisy features. The inclusion of multiple independent decision trees allows the model to ignore less informative variables, significantly enhancing robustness and prediction efficacy. Unlike KNN, which may be sensitive to noisy data, RF demonstrates inherent resilience to dataset noise and variability. By constructing models based on multiple trees, the impact of outliers or errors is mitigated, ensuring greater reliability in decision making. In summary, the preference for RF is justified by its ability to deliver robust and accurate predictive models, especially in complex environments and large datasets. Its resistance to overfitting, capacity to handle irrelevant features, and versatility relative to other algorithms make it a preferred choice, ensuring more reliable results and enhancing the model’s generalization capabilities.

As observed in the results, the proposed RF algorithm achieved the best outcomes in all analyzed parameters, with most metrics surpassing 90%. These results outperformed all other machine learning algorithms evaluated. This allows for the creation of an effective tool for classifying HCC patients. Moreover, the main determinants influencing the prognosis of HCC patients were identified. This tool aids in clinical practice for decision making by healthcare professionals, enhancing the quality of life for patients.

## 5. Conclusions

Meeting Milan criteria and BCLC classification are the variables with the greatest impact on the prognosis of patients with HCC in the developed predictive model. Both are valuable in clinical practice. Albumin levels emerge as an analytical variable that holds significant importance in the prognosis of HCC patients, and their combination with other analytical values may be interesting for the development of new prognostic biomarkers in HCC.

The proposed RF method achieves the best results in identifying the main prognostic factors of HCC at diagnosis and in the development of the predictive model. RF achieved superior results in all analyzed parameters, securing an AUC of 0.96 and standing out from the other proposed models. These results demonstrate that RF is useful and reliable for the analysis of prognostic factors in HCC diagnosis.

The development of a predictive prognostic model at the diagnosis of HCC aims to identify the factors that most influence patient mortality. This tool allows us to determine the prognosis of patients and, in the future, could help us personalize and optimize the management of patients at the time of HCC diagnosis. Therefore, it is essential to conduct the search for new prognostic factors and develop additional tools.

## Figures and Tables

**Table 1:** Baseline data collected from the study patients. ACLD: advanced chronic liver disease, HCV: Hepatitis C Virus, HBV: Hepatitis B Virus, BCLC: Barcelona Clinic Liver Cancer.

Sex	
Male	86.91%
Female	13.09%
Milan criteria	
No	62.83%
Yes	37.17%
Diagnostic by Screening	
No	52.88%
Yes	47.12%
ACLD	
Yes	87.96%
No	12.04%
Etiology	
Alcohol	32.46%
HCV	29.32%
HCV + Alcohol	13.61%
HBV	2.09%
Others	22.52%
BCLC	
Stage A	37.17%
Stage C	30.37%
Stage B	15.18%
Stage D	12.04%
Stage 0	5.24%

**Table 2:** Age at diagnosis and the most relevant analytical data collected from the study patients. Q1: first quartile, Q3: third quartile, IQR: interquartile range, AFP: alpha-fetoprotein, AST: aspartate aminotransferase, ALT: alanine aminotransferase.

	Age at Diagnosis	Bilirubin (mg/dL)	Albumin (g/dL)	Lymphocyte Count (Cell/mmc)	Platelet Count (Cell/mmc)	AFP (ng/mL)	AST (U/L)	ALT (U/L)
Median	68	1.005	3.7	1300	127,000	7.3	47	33
Q1	58	0.7	3.2	900	89,000	3.52	27	19
Q3	76.5	1.6	4.2	1900	184,500	83.35	72	60
IQR	18.5	0.9	1	1000	95,500	79.83	45	41

**Table 3:** The table shows the results of the mean values of accuracy, recall, specificity, precision, and *F*_1_ *score* obtained from the different ML models and RF method in the training phase. SVM: support vector machine, BLDA: Bayesian linear discriminant analysis, DT: decision tree, GNB: Gaussian naïve Bayes, KNN: K-nearest neighbors, RF: random forest.

Methods	Accuracy (%)	Recall	Specificity	Precision	*F*_1_ *Score*
SVM	88.35	88.73	88.24	87.72	88.08
BLDA	83.75	83.78	83.65	83.15	83.50
DT	86.56	86.65	86.82	85.83	86.39
GNB	82.15	82.13	82.06	81.57	81.91
KNN	91.44	91.36	91.41	91.39	91.37
RF	97.86	97.94	97.71	96.94	97.42

**Table 4:** The table presents the results of the mean values and standard deviation of *MCC*, AUC, *DYI* and Kappa score achieved from the different ML models and the RF method in the training phase. SVM: support vector machine, BLDA: Bayesian linear discriminant analysis, DT: decision tree, GNB: Gaussian naïve Bayes, KNN: K-nearest neighbors, RF: random forest.

Methods	*MCC*	AUC	*DYI*	Kappa
SVM	78.34	0.88	88.34	78.65
BLDA	74.31	0.84	83.72	74.56
DT	76.77	0.86	86.53	77.03
GNB	72.83	0.82	82.21	73.09
KNN	81.32	0.91	91.64	81.54
RF	86.62	0.97	97.63	86.91

**Table 5:** Results of the mean values and standard deviation of accuracy, recall, specificity, precision, and *F*_1_ *score* obtained from the different ML models and RF method in the study. SVM: support vector machine, BLDA: Bayesian linear discriminant analysis, DT: decision tree, GNB: Gaussian naïve Bayes, KNN: K-nearest neighbors, RF: random forest.

Methods	Accuracy	Recall	Specificity	Precision	*F*_1_ *Score*
SVM	86.82 ± 0.67	86.92 ± 0.62	86.72 ± 0.63	86.20 ± 0.64	86.56 ± 0.65
BLDA	82.31 ± 0.82	82.40 ± 0.83	82.21 ± 0.82	81.72 ± 0.83	82.06 ± 0.84
DT	85.05 ± 0.71	85.15 ± 0.68	84.95 ± 0.69	84.45 ± 0.71	84.80 ± 0.69
GNB	80.73 ± 0.92	80.83 ± 0.97	80.64 ± 0.96	80.16 ± 0.97	80.49 ± 0.98
KNN	90.06 ± 0.58	90.17 ± 0.56	89.95 ± 0.58	89.42 ± 0.59	89.79 ± 0.58
RF	95.94 ± 0.35	96.05 ± 0.32	95.83 ± 0.36	95.26 ± 0.37	95.65 ± 0.38

**Table 6:** Results of the mean values and standard deviation of MMC, AUC, *DYI*, and Kappa obtained from the different ML models and RF method in the study. SVM: support vector machine, BLDA: Bayesian linear discriminant analysis, DT: decision tree, GNB: Gaussian naïve Bayes, KNN: K-nearest neighbors, RF: random forest, *MCC*: Matthews’ correlation coefficient, AUC: area under the curve, *DYI*: degenerated Youden index.

Methods	*MCC*	AUC	*DYI*	Kappa
SVM	77.04 ± 0.58	0.87 ± 0.02	86.82 ± 0.65	77.29 ± 0.57
BLDA	73.03 ± 0.61	0.82 ± 0.02	82.31 ± 0.81	73.27 ± 0.63
DT	75.47 ± 0.51	0.85 ± 0.02	85.05 ± 0.68	75.72 ± 0.52
GNB	71.64 ± 0.75	0.81 ± 0.02	80.73 ± 0.93	71.88 ± 0.76
KNN	79.91 ± 0.48	0.90 ± 0.02	90.06 ± 0.57	80.18 ± 0.49
RF	85.13 ± 0.27	0.96 ± 0.02	95.94 ± 0.36	85.41 ± 0.26

## Data Availability

The datasets used and/or analyzed during the present study are available from the corresponding author upon reasonable request.
